# Disseminated bartonellosis and EBV reactivation in an adolescent treated with upadacitinib

**DOI:** 10.1016/j.bjid.2025.104536

**Published:** 2025-05-01

**Authors:** Vincent Pargny, Emilie Delugre, Raphael Janela, Soumaya Skalli, Abdeljalil Zeggay, Hortense Petat

**Affiliations:** a Pediatrics Department, Rouen University Hospital, France; b Infectious Diseases Department, Rouen University Hospital, France; c Dermatology Department, Rouen University Hospital, France; d Department of Microbiology, Rouen University Hospital, France; e Univ Rouen Normandie, Université de Caen Normandie, INSERM, Normandie Univ, France

**Keywords:** Bartonellosis, Immunodepression, Upadacitinib, Pediatrics

## Abstract

**Background:**

*Bartonella henselae* is responsible for disseminated infections in immunocompromised patients. Upadacitinib is a JAK inhibitor used since 2021 for the treatment of severe atopic dermatitis in adolescents over 12-years. We report here the case of disseminated Bartonellosis in a 15-year-old male treated with upadacitinib.

**Case presentation:**

This patient presented with fever and abdominal pain. Laboratory tests showed inflammation, rhabdomyolysis and acute renal failure. Imaging studies revealed lymphadenopathies, hepatosplenic, renal and pulmonary nodules, pleural and peritoneal effusions and spleen abscesses. *Bartonella henselae* serology was positive for IgG and IgM. Treatment with doxycycline resolved the symptoms. We detected a concomitant EBV reactivation.

**Conclusions:**

*Bartonella henselae* infection should be suspected in immunocompromised patients presenting with fever of unknown origin. Upadacitinib is responsible for rapid immunodepression, which can lead to viral reactivation and severe bacterial infections. Randomized controlled trials are needed to establish a consensus on treatments for the various forms of Bartonellosis.

## Highlights

• Bartonella henselae can cause severe infections in immunocompromised patients.
• Bartonella henselae infection should be suspected in cases of fever of unknown origin.
• Upadacitinib (JAK inhibitor) increases the risk of severe bacterial infections, opportunistic infections and viral reactivation.
• Upadacitinib increases the risk of CPK elevation.

## Background

Upadacitinib is an oral reversible JAK inhibitor that has been recommended for use since 2021 in France in the treatment of moderate to severe atopic dermatitis in patients over 12-years requiring systemic treatment.^1^

*Bartonella henselae* is a ubiquitous pathogen transmitted by exposure to cats, particularly those infested with fleas. The ability to cause acute or chronic infections and vascular proliferative or suppurative granulomatous manifestations is a remarkable feature of *Bartonella* spp. The severity of clinical manifestations correlates with the patient's immune status. *Bartonella* spp. are responsible for a number of diseases including Cat-Scratch Disease (CSD), chronic lymphadenopathy, trench fever, endocarditis, bacillary angiomatosis, peliosis hepatis and neurological disorders.^2^

We report here the case of a 15-year-old adolescent who developed disseminated Bartonellosis under treatment with Upadacitinib for atopic dermatitis.

## Case presentation

We present the case of a 15-year-old male, who was referred to the pediatric emergency department of our tertiary center for suspected appendicitis due to abdominal pain associated with vomiting and fever for three days (D1 is the first day of fever), with marked asthenia and anorexia. He was treated for one month with Upadacitinib 15 mg daily for severe atopic dermatitis.

Biological tests showed a lymphopenia of 1.24 G/L, a hepatic cytolysis associated with rhabdomyolysis (CPK 45 919 IU/L) and acute renal failure (creatinine 132 µmoL/L; urea 7.6 mmoL/L). Ceftriaxone-based antibiotic therapy was initiated in this setting of hyperthermia and biological inflammatory syndrome (CRP 150 mg/L). Upadacitinib was suspended. Abdominal ultrasound found no evidence of appendicitis or pancreatitis.

The patient was hospitalized in pediatric intensive care to control rhabdomyolysis and acute renal failure. Abdominal ultrasound at Day 2 revealed globular left kidney, with 2 hypoechoic areas compatible with foci of nephritis. Ceftriaxone was thus continued. Hyperthermia persisted, between 39 °C and 40 °C, and the CRP peaked at 212 mg/L at Day 7.

Nasal PCR test, cytobacteriological urinary analysis and blood cultures were negative. PCR tests for leptospirosis were negative. Serologies for HSV types 1 and 2, HIV, HAV, HBV, HCV, HEV, CMV and toxoplasmosis were negative. Serologies for EBV, VZV, HHV6, Parvovirus B19 and *Mycoplasma pneumoniae* showed past infections. EBV viral load was 4.67 log_10_copies/mL. Quantiferon test was negative. Metabolic tests were normal. Antinuclear antibodies were negative. Toxicological tests were negative.

**Fig. 1:**
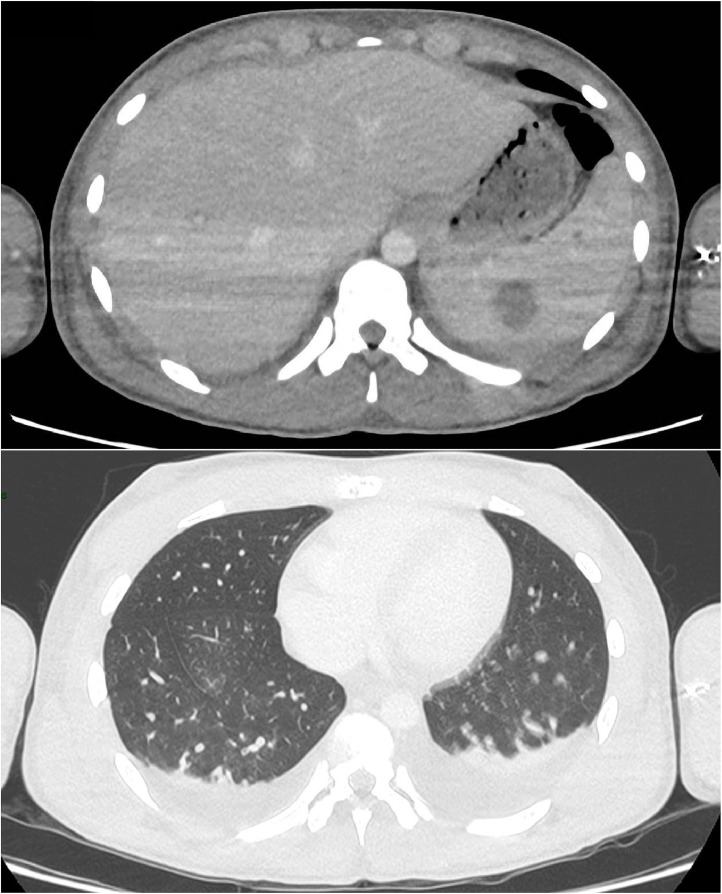
Hepatosplenic and pleuropulmonary lesions. CT scan performed at Day 10. Adenomegalia of the celiac region and hepatic hilum. Hepatomegaly with one lesion of the left liver. Multiple splenic lesions.

At Day 9, rhabdomyolysis and renal failure improved, so the patient was transferred to the pediatric unit. CT scan performed at Day 10 (Fig. 1) showed extensive lymph node formation in the thorax, with a bilateral pleural effusion. The kidneys, liver and spleen were hypertrophied with multiple hypodense nodular lesions. There are also multiple adenomegalia of the coeliac region and an intraperitoneal effusion.

A PET-CT scan performed at Day 16 suggested a lymphoproliferative syndrome: intense hypermetabolism of multiple spontaneously hypodense intrahepatic bi-lobar and intra-splenic lesions and more moderate hypermetabolism of several supra- and subdiaphragmatic lymphadenopathies. The lymphocyte immunophenotyping was normal. We did not perform a biopsy because of the difficulty of accessing lymphadenopathies.

Asthenia and abdominal pain rapidly resolved. Fever subsided at Day 16. We changed antibiotic therapy to ceftriaxone *plus* metronidazole on Day 12 in view of the hepatosplenic lesions considered as abscesses. We started antibiotic oral relay at D19 with amoxicillin-clavulanic acid. Ultrasound examinations (at Days 19 and 24) revealed a reduction of hepatosplenic lesions and lymphadenopathies. Radiologists described hepatosplenic micronodular miliary, with “target lesions” suggestive of granulomatosis.

*Bartonella henselae* serology (IFA; SELAFA CERBA – 10/12 av. Roland Moréno – ZAC des Epineaux – 95740 Frépillon – France) was positive with an antibody titer compatible with a recent infection (IgM 1:96, significance threshold 1:48; IgG negative) but blood PCR was negative (house RT-PCR, targets: ITS3 and pap31; French National Reference Center of *Rickettsia, Coxiella* and *Bartonella* – 19/21 Bd Jean Moulin – 13385 Marseille – France). The antibody titer remained stable over a period of six weeks. The patient described close contact with a flea-infested 2-year-old cat. Although the biological diagnosis cannot be confirmed, the possible exposure, the immunodepression and the compatible clinical presentation led us to strongly suspect disseminated Bartonellosis and to treat the patient. At Day 33, amoxicillin-clavulanic acid was replaced by doxycycline 100 mg twice daily for 14 days.

At Day 58, the patient's health had fully recovered. The CT scan showed almost complete regression of visceral lesions and abdominal adenopathy, with the persistence of a small splenic lesion at the site of the largest abscess. EBV viral load decreased to 3.29 log_10_copies/mL.

## Discussion

We report a case of disseminated Bartonellosis with rhabdomyolysis and EBV reactivation in a 15-year-old male treated with upadacitinib for atopic dermatitis.

Safety studies of upadacitinib show a dose-dependent elevation of CPK in treated patients. These patients were mostly asymptomatic, and some had vigorous physical activity prior to blood sampling.^3^ A few authors reported cases of *Bartonella* myositis (with elevated CPK): in a 14-year-old girl with Turner syndrome, a 26-month-old healthy boy, an immunocompetent 12-year-old girl, and an immunocompromised 66-year-old patient living with Human Immunodeficiency Virus (HIV).^4^, ^5^, ^6^ Moreover, sepsis, bacteremia and EBV infection are frequent causes of rhabdomyolysis.^7^ In this case, rhabdomyolysis could be attributed to the combination of upadacitinib treatment, EBV reactivation and severe bacterial infection.

Upadacitinib causes a dose-dependent increased risk of severe and opportunistic infections. The most common severe infection reported with upadacitinib treatment is herpes zoster. The main opportunistic infections reported are oesophageal candidiasis and eczema herpeticum. Thus, viral reactivations (HSV and VZV) are common under upadacitinib.^3^ This may explain the EBV reactivation in our patient. These factors may point to the immunosuppressive role of upadacitinib in explaining the severity of this case. Furthermore, the risk of severe or opportunistic infections attributable to upadacitinib does not appear to be influenced by the duration of treatment which explains why this case developed a severe infection after only 1 month of treatment.^3^

Indeed, numerous studies show the increased severity of *Bartonella* infections in cases of immunodepression, especially in HIV patients and kidney transplant recipients.^8^^,^^9^ For these patients, *Bartonella* species can produce a broad array of manifestations, including bacillary angiomatosis, peliosis hepatis, splenitis, osteomyelitis, and bacteremia.^8^ Tram *et al*. reported the case of a Caucasian 7-year-old girl, followed in pediatric nephrology for an idiopathic nephrotic syndrome since the age of five, admitted for fever of unknown origin while receiving mycophenolate mofetil.^10^ This child's Bartonellosis was similar to the case we report: fever, visceral damages (hepatosplenic nodules) and multiple lymphadenopathies. Hepatosplenic forms of the disease are rare in immunocompetent subjects, and appear to be more frequent in children and adolescents.^11^^,^^12^ To our knowledge, the scientific literature does not describe any other cases of disseminated Bartonellosis occurring in patients treated with JAK inhibitors.

Biological diagnosis of bartonellosis is not easy. Serology alone is not sufficient, and must be supported by a compatible clinical picture and/or PCR confirmation. The specificity of IgM (IFA) is high (>90 %), but co-infection with EBV can lower it.^13^^,^^14^ Blood PCR has a sensitivity of 35 %, except in cases of endocarditis in which it can reach 90 %. An early PCR on a lymph node aspiration is more sensitive but we were unable to perform it in our case due to difficulties in accessing mediastinal lymphadenopathies.^9^^,^^15^

Antibiotic treatment for Bartonellosis is non-consensual and depends on the various forms the disease can take.^16^ Molecules with intracellular activity are used: macrolides, cyclins and quinolones. Rifampin is used in combination in neurological or ophthalmological disease forms. In France, CSD is treated with a 5-day course of azithromycin, but the treatment of atypical or disseminated Bartonellosis is non-consensual and empirical, due to the lack of evidence-based data.^2^^,^^15^ In our case, we opted for a 14-day course of doxycycline 200 mg/d, in line with the recommended treatment for *Bartonella* liver abscesses.^15^

To our knowledge, this case report is the first to describe disseminated Bartonellosis and EBV reactivation in a patient receiving upadacitinib. This case description highlights the importance of looking for Bartonellosis in cases of fever of unknown origin, multiple lymphadenopathies and hepatosplenic damages, especially in immunocompromised patients. Fever in a patient receiving upadacitinib should prompt a search for viral reactivation. Randomized controlled trials are needed to establish a consensus on treatments for the various forms of Bartonellosis.

## Authors’ contributions

VP: Data curation; writing-original draft preparation.

ED: Data curation; writing-original draft preparation.

RJ: Methodology; writing-reviewing and editing.

SS: Resources; writing-reviewing and editing.

AZ: Conceptualization; methodology; writing-reviewing and editing.

HP: Supervision; conceptualization; methodology; writing-reviewing and editing.

All authors approved the final manuscript as submitted and agree to be accountable for all aspects of the work.

## Conflicts of interest

The authors declare no conflicts of interests.
